# Supplementary material for: LIMT is a novel metastasis inhibiting lncRNA suppressed by EGF and downregulated in aggressive breast cancer
Source: EMBO Mol Med. 2016 Aug 3;8(9):1052–64. doi: 10.15252/emmm.201606198 (PMC5009810; doi:10.15252/emmm.201606198)
Supplement: Supplementary file 1 — Appendix [file EMMM-8-1052-s001.pdf]

Appendix

Table of content

Appendix Figure S1: Expression of LIMT in breast cancer specimens associates with disease parameters.

Appendix Table S1: A list of human cells lines used in the study along with details on contents of culture media.

Appendix Table S2: Sequences of primers used for real-time qPCR.

Appendix Table S3: Sequences of siRNAs used to knock-down lncRNAs.

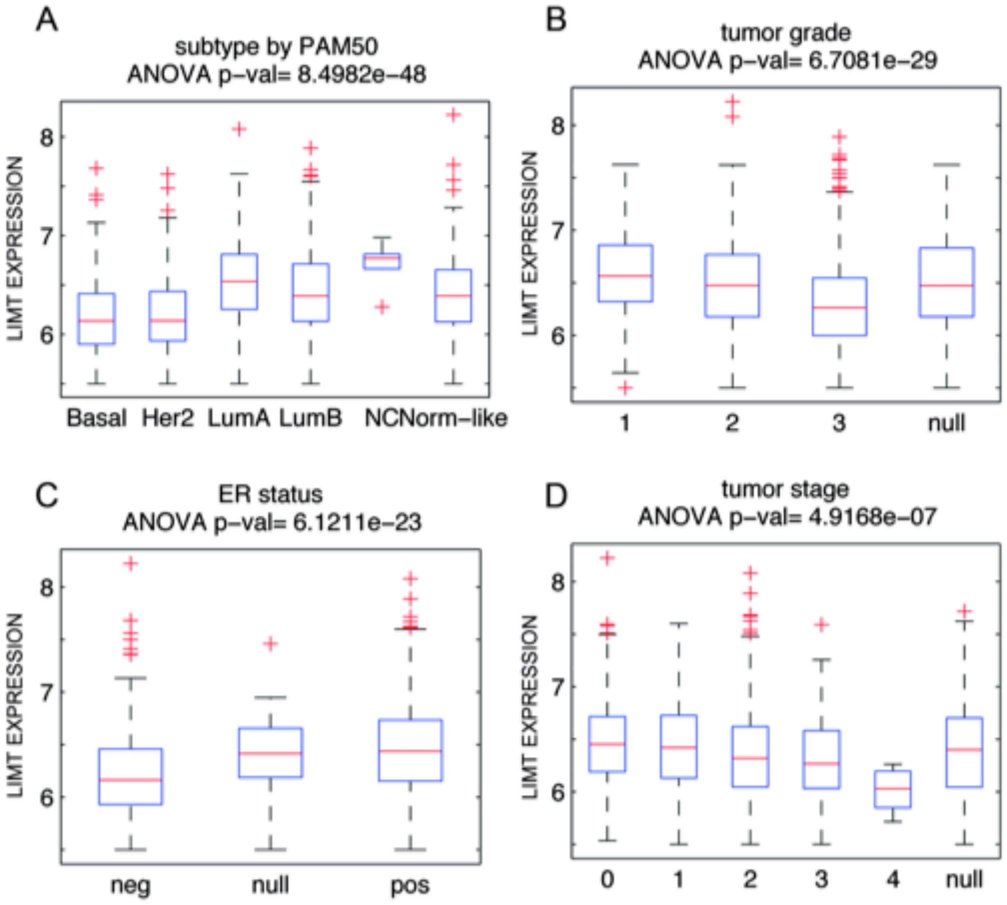

Appendix Figure S1. Expression of LIMT in breast cancer specimens associates with disease parameters. The expression of LIMT is shown in respect to the following parameters: PAM50 subtypes (**A**), tumor grade (**B**), ER status (**C**) and tumor stage (**D**). The METABRIC dataset was used to analyze the abundance of LIMT's transcripts in breast cancer specimens of different molecular subtypes, which were determined by using the PAM50 classifier. To evaluate differences in LIMT expression between the groups we used a one-way analysis of variance (ANOVA).

Appendix Table S1. Human cell lines

Cell Line	ATCC #	Type	Medium	FBS	Gln	Pen / Strep
H1299	NCI-H1299	Lung	RPMI	10% FBS	1% Gln (0.2 mM)	1% P/S
A549	A549-atcc	Lung	DMEM	10% FBS	1% Gln (0.2 mM)	1% P/S
PC9	PC9	Lung	RPMI	10% FBS	1% Gln (0.2 mM)	1% P/S
WI-38	WI-38	Lung	DMEM high glucose/MEM	10% FBS	1% Gln (0.2 mM)	1% P/S
Calu-1	Calu-1	Lung	RPMI/McCoy's 5a Medium Modified	10% FBS	1% Gln (0.2 mM)	1% P/S
Calu-6	Calu-6	Lung (unknown, Probably lung)	RPMI/ MEM	10% FBS	1% Gln (0.2 mM)	1% P/S
H1703	NCI-H1703	Lung	RPMI	10% FBS	1% Gln (0.2 mM)	1% P/S
H23	NCI-H23	Lung	RPMI	10% FBS	1% Gln (0.2 mM)	1% P/S
H292	NCI-H292	Lung	RPMI	10% FBS	1% Gln (0.2 mM)	1% P/S
IMR90	IMR90	Lung	DMEM high glucose/MEM	10% FBS	1% Gln (0.2 mM)	1% P/S
B2B	B2B	Lung	RPMI/BEBM	10% FBS	1% Gln (0.2 mM)	1% P/S
H460	NCI-H460	Lung	RPMI	10% FBS	1% Gln (0.2 mM)	1% P/S
H520	NCI-H520	Lung	RPMI	10% FBS	1% Gln (0.2 mM)	1% P/S
HLE	HLE	Liver	DMEM	10% FBS	0	0
Huh-7	Huh-7	Liver	DMEM	10% FBS	1% Gln (0.2 mM)	1% P/S
HLF	HLF	Liver	DMEM	5% FBS	0	0
HepG2	HepG2	Liver	RPMI/MEM	10% FBS	1% Gln (0.2 mM)	0
MM20	MM20	Skin	RPMI	10%FBS	1% Gln (0.2 mM)	0
MM71	MM71	Skin	RPMI	10%FBS	1% Gln (0.2 mM)	0
MM47	MM47	Skin	RPMI	10%FBS	1% Gln (0.2 mM)	0
HeLa	HeLa	Cervix	DMEM/MEM	10%FBS	1% Gln (0.2 mM)	0
SKBR3	SKBR3	Breast	McCoy's 5A	10% FBS	1% Gln (0.2 mM)	0
T47D	T47D	Breast	DMEM/RPMI	10% FBS	1% Gln (0.2 mM)	0
BXPC3	BXPC3	Pancreas	RPMI	10%FBS	1% Gln (0.2 mM)	0
A-673	A-673	Muscle	DMEM	10% FBS	0	0
1FF	1°FF	Skin	DMEM	10%FBS	1% Gln (0.2 mM)	0
HaCaT	HaCaT	Skin	DMEM (high glucose)	10%FBS	1% Gln (0.2 mM)	0
RKO	RKO	Colon	DMEM/MEM	10% FBS	0	0
HCT-116	HCT-116	Colon	McCoy's 5A	10%FBS	1% Gln (0.2 mM)	0
AGS	AGS	Stomach	F12-K	10% FBS	0	0

Appendix Table S1. Human cell lines (continued)

Cell Line	ATCC #	Type	Medium	FBS	Gln	Pen / Strep
MKN-45	MKN-45	Stomach	RPMI	10% FCS	0	0
SAOS-2	SAOS-2	Bone	DMEM/McCoy's 5a Medium Modified	10% FBS/15% FBS	1% Gln (0.2 mM)	0
U2OS	U2OS	Bone	DMEM/McCoy's 5a Medium Modified	10% FBS	1% Gln (0.2 mM)	0
MOLT-4	MOLT-4	Blood	RPMI	10% FBS	1% Gln (0.2 mM)	0
CCRF-CEM	CCRF-CEM	Blood/Peripheral	RPMI	10% FBS	1% Gln (0.2 mM)	0
K562	K562	Blood /Bone Marrow	RPMI/Iscove's Modified Dulbecco's Medium	10% FBS	1% Gln (0.2 mM)	1% P/S
Jurkat	Jurkat	Blood	RPMI	10% FBS	1% Gln (0.2 mM)	0
NT2	NT2	Testis	DMEM	10%FBS	0	0
HEK293	HEK293	Kidney	DMEM/MEM	10%FBS	1% Gln (0.2 mM)	0
Tet-21	Tet-21	Brain	RPMI	10% Tet-approved FBS	1% Gln (0.2 mM)	0
MCF10A	MCF10a	Breast	special see new excel sheet/MEBM	0	0	0
MCF7	MCF7	Breast	DMEM/MEM	10% FBS	1% Gln (0.2 mM)	0
MDA-MB-231	MDA-MB-231	Breast	DMEM/Leibovitz's L-15 Medium	10% FBS	1% Gln (0.2 mM)	0

Appendix Table S2. Oligonucleotide primers

**Gene**	**Forward primer**	**Reverse primer**
LIMT	GGGCAGGCCTAGTTTGACTC	AGTCACTCCCCCTTCCAGTC
LOC388796	GGATGATGTAGGGGAAGCAA	ACAGCCACTGAAAGCATGTG
GAPDH	GTGAAGGTCGGAGTCAACG	TGAGGTCAATGAAGGGGTC
B2M	GGCATTCCTGAAGCTGAC	TCTTTGGAGTACGCTGGATAG
Egr1	AGCCCTACGAGCACCTGA	GGTTTGGCTGGGGTAACTG

Appendix Table S3. siRNA oligonucleotides and shRNA sequences

Gene Symbol	GENE ID	Gene Accession	GI Number	Sequence
EGFR	1956	NM_201283	41327733	CAAAGUGUGUAACGGAAUA
EGFR	1956	NM_201283	41327733	CCAUAAAUGCUACGAAUAU
EGFR	1956	NM_201283	41327733	GUAACAAGCUCACGCAGUU
EGFR	1956	NM_201283	41327733	CAGAGGAUGUUCAAUAACU
PLK1	5347	NM_005030	34147632	GCACAUACCGCCUGAGUCU
PLK1	5347	NM_005030	34147632	CCACCAAGGUUUUCGAUUG
PLK1	5347	NM_005030	34147632	GCUCUUCAAUGACUCAACA
PLK1	5347	NM_005030	34147632	UCUCAAGGCCUCCUAAUAG
LOC282997	282997	NR_026932	223941947	UCACAUUCUACCUGGCAUU
LOC282997	282997	NR_026932	223941947	GUGGCCAGUAUCUGAAUUA
LOC282997	282997	NR_026932	223941947	GAAAUAGGGUUGUCGUCCU
LOC282997	282997	NR_026932	223941947	GGGUAGGCAUCCCGAGUUC
NEAT1	283131	NR_028272	255306270	GGGAAGUAGUCUCGGGUAU
NEAT1	283131	NR_028272	255306270	GCAAACAAUUACUGUCGUU
NEAT1	283131	NR_028272	255306270	UGAAAUAACUUGGGGCGUU
NEAT1	283131	NR_028272	255306270	AAAUUGAGCCUCCGGUCAU
LINC01089	338799	NR_002809	223671895	CUAUAGGGCUUGAGUUAUU
LINC01089	338799	NR_002809	223671895	GGGCAGGCCUAGUUUGACU
LINC01089	338799	NR_002809	223671895	CAUUCAUGUCAGCAGUUAA
LINC01089	338799	NR_002809	223671895	CCACGUUAGCAUUCGCAUU
LOC642852	642852	NR_026943	223972655	CGUCUUUGCUUUCCGGAGA
LOC642852	642852	NR_026943	223972655	GCACUCGGUGGCUCGGAAA
LOC642852	642852	NR_026943	223972655	GAGCUGUCCCUUCGCGGAA
LOC642852	642852	NR_026943	223972655	CAAGUGACCAAGUCGUGAA
LOC388796	388796	NR_015366	224549035	GGAUCUGGGUUUGCUGAUA
LOC388796	388796	NR_015366	224549035	CUUGGAUGAUGUAGGGGAA
LOC388796	388796	NR_015366	224549035	GUCCUAAGACCAUGGAGUU
LOC388796	388796	NR_015366	224549035	GCACAAGAUUCGGGGUCCA
LINC00472	79940	NR_026807	223468561	GCCUAAACUAAGACGGAUA
LINC00472	79940	NR_026807	223468561	CAACUUGCGAACCGGGAAA
LINC00472	79940	NR_026807	223468561	CUUUAAAGUGUGUCGACAU
LINC00472	79940	NR_026807	223468561	GGGCUGCGGAUUUCAGGUA
LOC344595	344595	NR_028302	255683376	AUCCUUAAGAGGUGAAUUU
LOC344595	344595	NR_028302	255683376	AAUCCAGCAUCGCGUGGUA
LOC344595	344595	NR_028302	255683376	CGAGGGCUGCAGAGAGCAU
LOC344595	344595	NR_028302	255683376	CCAGAUAGAAGACCGGAGC
HSPC072	29075	NR_026884	223718125	CCACAAUCGUUCCAGCCAA
HSPC072	29075	NR_026884	223718125	CUAAGGAUGUACUGCUGAU
HSPC072	29075	NR_026884	223718125	AGUGCAGCUACUUUGGAAA
HSPC072	29075	NR_026884	223718125	UUAGAAUGGCAGAAAUCGA
HYMAI	57061	NR_002768	84872052	CUUACAACCUGGCGCUCUA
HYMAI	57061	NR_002768	84872052	GUAAAACACAGACGGAGAA
HYMAI	57061	NR_002768	84872052	UGGAAAUGCUUUAUCGCCU
HYMAI	57061	NR_002768	84872052	GUCCACAAGCAGACGUGUA
**Gene Symbol**	**GENE ID**	**Gene Accession**	**shRNA Name**	**Sequence**
LINC01089	338799	NR_002809	sh1483	GCACAACTGCCTCTCAATAAA
LINC01089	338799	NR_002809	sh716	CATTCATGTCAGCAGTTAA
