# Supplementary material for: LIMT is a novel metastasis inhibiting lncRNA suppressed by EGF and downregulated in aggressive breast cancer
Source: EMBO Mol Med. 2016 Aug 3;8(9):1052–64. doi: 10.15252/emmm.201606198 (PMC5009810; doi:10.15252/emmm.201606198)
Supplement: Supplementary file 2 — Expanded View Figures PDF [file EMMM-8-1052-s002.pdf]

Expanded View Figures

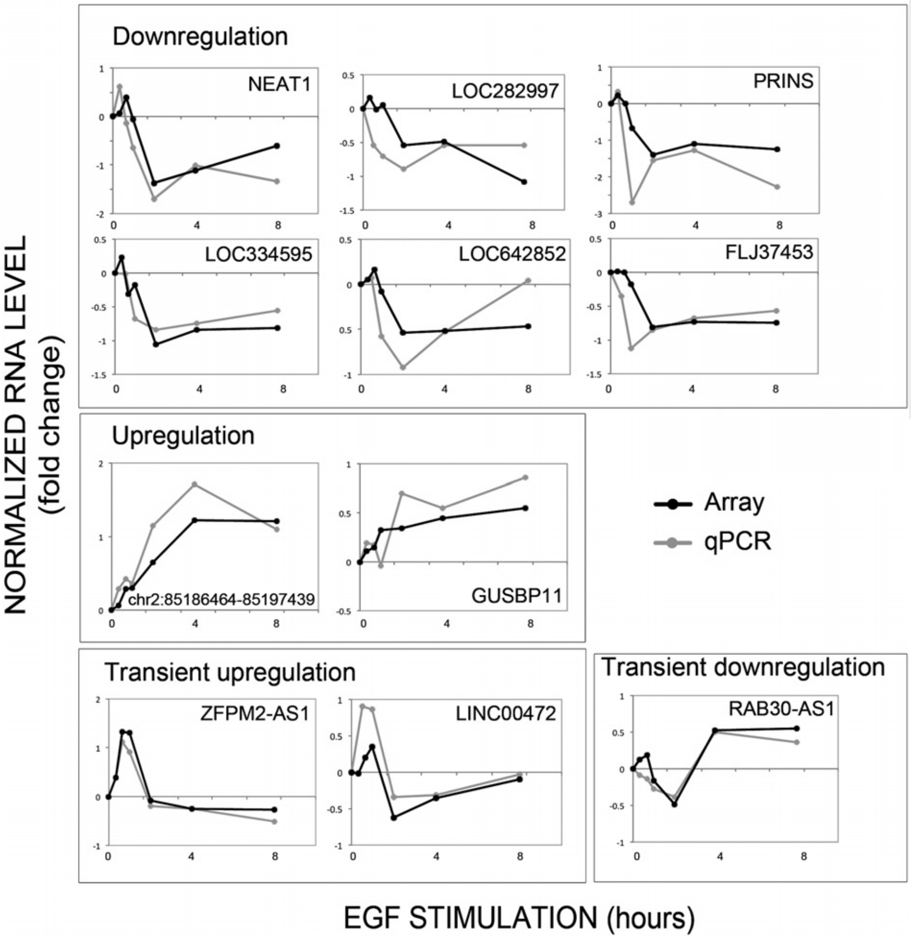

Figure EV1. Dynamic changes of lncRNAs in response to stimulation of mammary cells with EGF.
RNA from the samples presented in Fig 1B was converted into cDNA, and levels of the indicated lncRNAs were measured using real-time qPCR. RNA was collected at 0, 20, 40, 60, 120, 240, and 480 min after stimulation with EGF (10 ng/ml). Expression values are presented as log2 fold change relative to time zero, and they are normalized to beta-2-microglobulin's mRNA. Each panel depicts expression profiles determined using both microarrays (see Fig 1; black) and qPCR (gray). The lncRNAs are allocated into four clusters according to their expression profile: downregulation, upregulation, transient downregulation, and transient upregulation.

 © 2016 The Authors

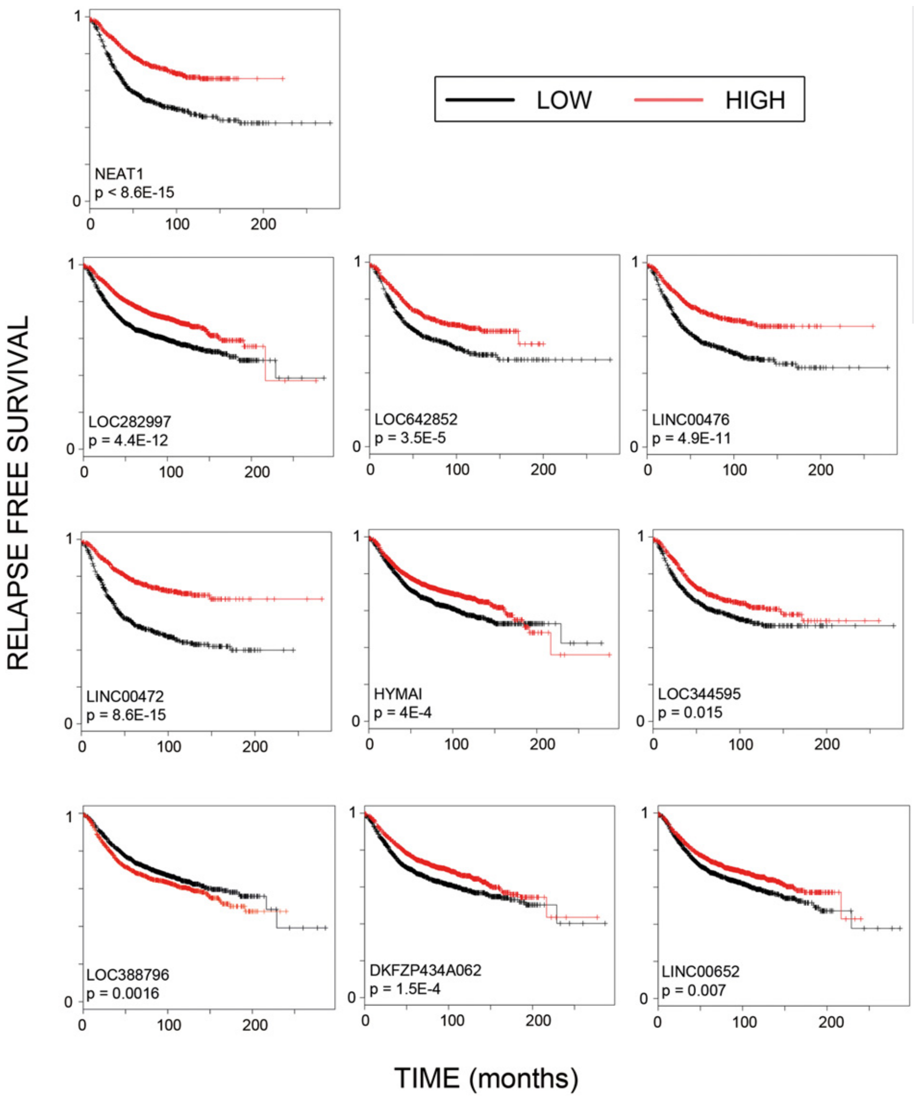

Figure EV2. Expression levels of EGF-regulated lncRNAs associate with clinical outcome of breast cancer patients.
Kaplan–Meier survival plots, depicting expression levels of specific lncRNAs and clinical outcomes of breast cancer patients. The plots were generated for EGF-regulated lncRNAs showing significant correlation between their expression levels and the respective patients' relapse-free survival time, as determined in at least one dataset (METABRIC and/or KM-plotter). The adjusted *P*-value of each plot appears below the name of the lncRNA.

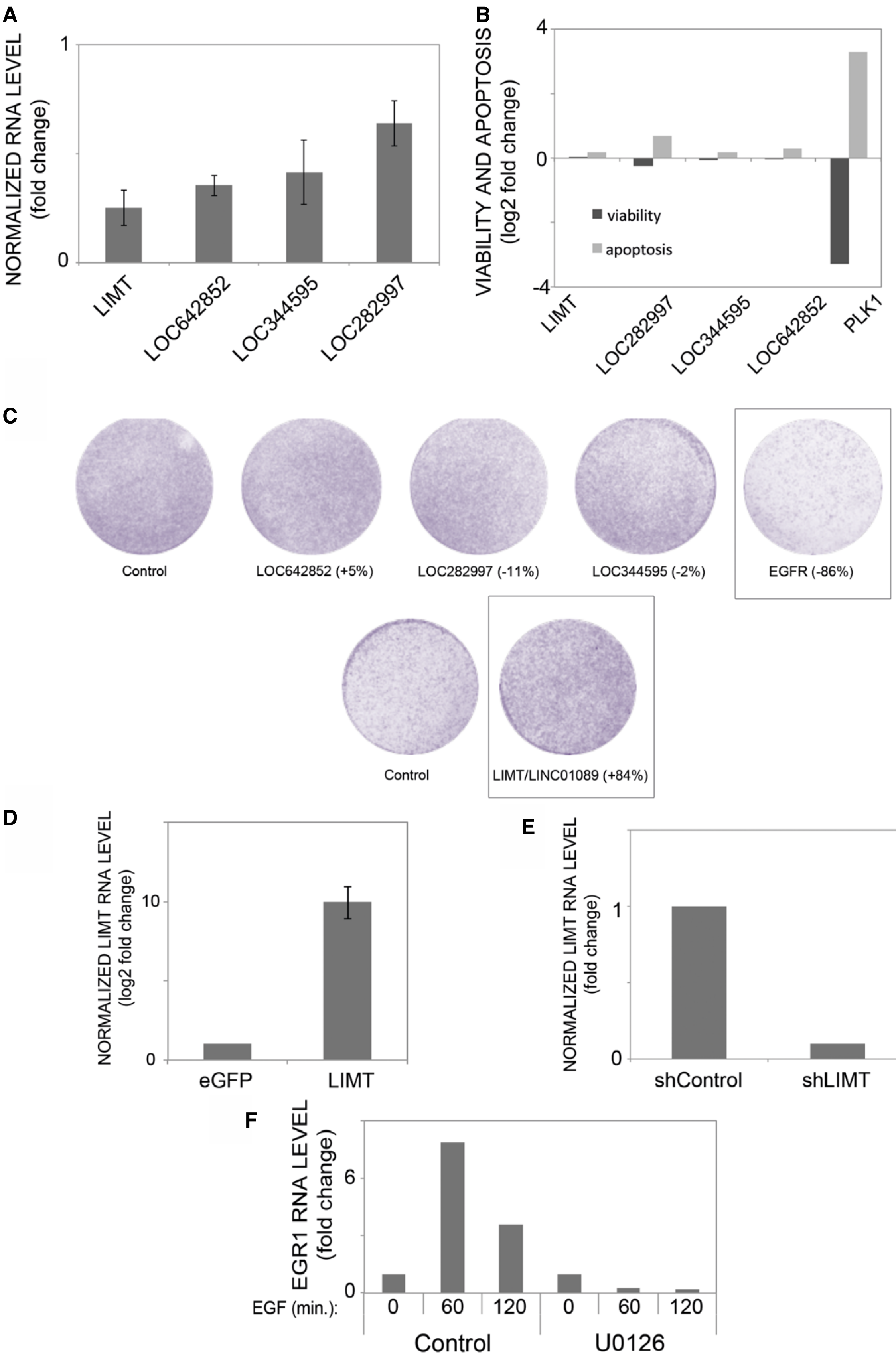

Figure EV3.

 © 2016 The Authors

Figure EV3. An siRNA screen identifies LIMT as an ERK-dependent negative regulator of mammary cell migration.

A MCF10A cells were transfected with siRNA pools specific for the indicated lncRNAs. Forty-eight hours later, cells were harvested, RNA converted to cDNA, and analyzed using RT–qPCR. Knockdown efficiency of each lncRNA-specific siRNA is presented relative to transfection with control siRNAs. Shown are means ± SD of triplicates. The experiment was repeated twice.

B MCF10A cells were transfected with lncRNA-specific (or control) siRNAs, and the effects on viability (WST-1 assay) and apoptosis were measured. For each assay, the fold change between lncRNA-specific siRNA and control siRNA was measured. Transfection with PLK1-specific siRNAs was used as reference. The viability and apoptosis assays were repeated twice.

C MCF10A cells were transfected with lncRNA-specific (or control) siRNAs. Forty-eight hours later, cells were seeded on the upper faces of Transwell migration chambers and left to migrate for 20 h in full medium. Cells on the lower faces of the chambers were then fixed, stained, and imaged for quantification of migration. *EGFR*-specific siRNAs were used as positive control for inhibition of migration. Shown are images of the stained cells. Numbers in brackets indicate the effect of knockdown on migration (relative to control siRNAs). Shown are results of one experiment, which was repeated twice.

D MCF10A cells were stably transfected with plasmids overexpressing the LIMT transcript, or eGFP as control. RNA levels of LIMT were measured using RT–qPCR. Shown are means ± SD of triplicates.

E MCF10A cells were stably transfected with a LIMT-specific shRNA plasmid or with a control shRNA plasmid. RNA level of LIMT was measured using RT–qPCR.

F MCF10A cells were treated as in Fig 3C. Sixty and 120 min after the addition of EGF, cells were lysed and purified RNA was used to determine EGR1's transcript levels using real-time qPCR. Expression values are presented as fold change relative to time zero and are normalized to beta-2-microglobulin.

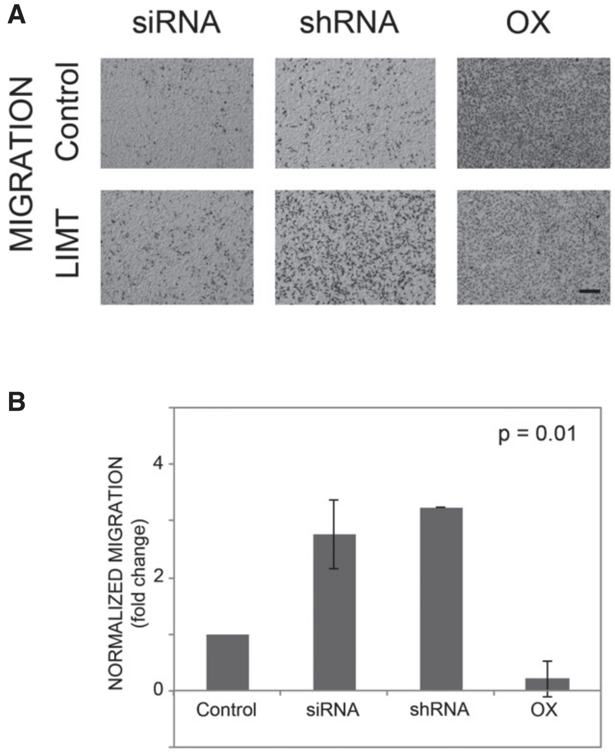

Figure EV4. LIMT inhibits migration of MDA-MB-231 cells.

A MDA-MB-231 cells were transfected with LIMT-specific siRNAs (or with control siRNA), and their migration (left panels) was measured using Transwell migration chambers. Cells that migrated to the lower face of the chambers were fixed, stained, and imaged. Likewise, cells were stably transfected with plasmids encoding for shRNAs against LIMT (middle panels) or with plasmids driving overexpression (OX) of either LIMT or eGFP (Control, right panels). Bar, 0.2 mm.

B Quantification of cell migration upon lncRNA knockdown using either siRNA or shRNA, and overexpression (OX). *P*-values of one-way ANOVA are presented. Values represent means ± SD of triplicates.

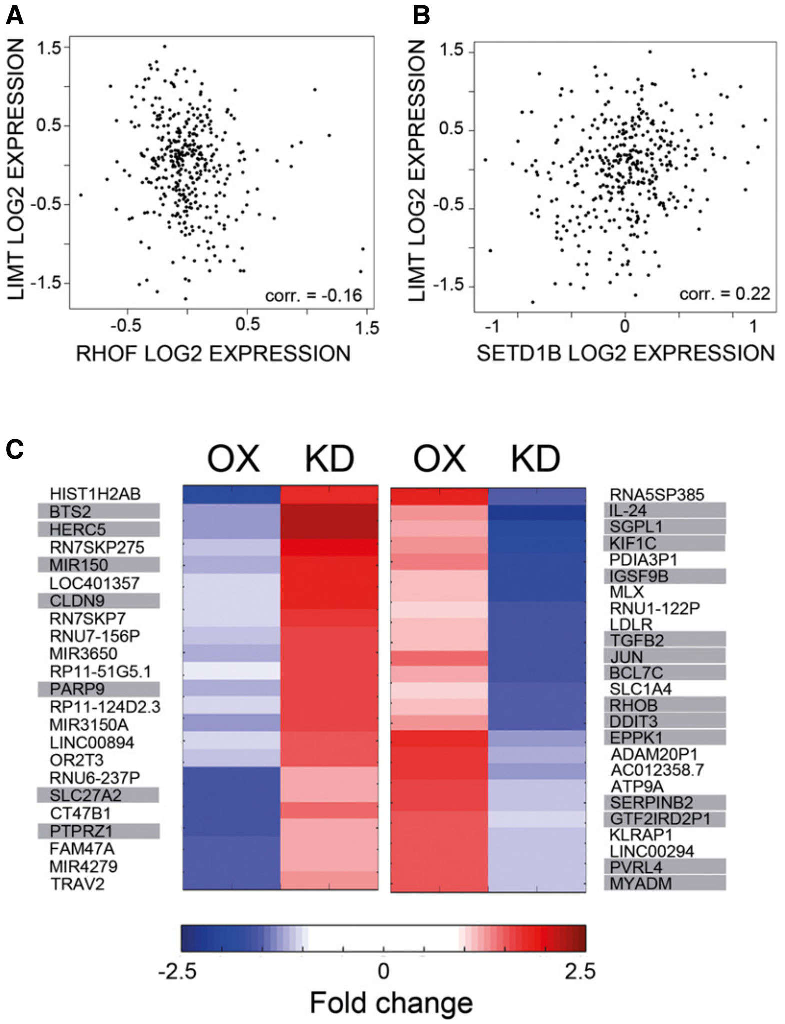

Figure EV5. Manipulation of LIMT levels affects the expression of coding and noncoding genes in trans.

A, B Using Agilent microarrays, the expression of *LIMT* was assayed in all breast cancer specimens of the Oslo2 study (N = 381). The log2 expression of *LIMT* in patients was plotted against the log2 expression of its neighboring coding genes, RHOF (A) and SETD1B (B). Pearson correlation values are indicated.

C LIMT was stably overexpressed or transiently knocked down (using siRNAs) in MCF10A cells. RNA was processed and hybridized to Affymetrix gene expression microarrays. Shown are expression levels of 48 selected genes, which showed a significant fold change of at least 1.5 under at least one of the conditions. Genes are presented in two clusters according to their response to manipulation of LIMT (OX, overexpression; KD, knockdown). Highlighted are genes that have previously been implicated in cancer progression or regulation of cell migration.

 © 2016 The Authors
