# Supplementary material for: LIMT is a novel metastasis inhibiting lncRNA suppressed by EGF and downregulated in aggressive breast cancer
Source: EMBO Mol Med. 2016 Aug 3;8(9):1052–64. doi: 10.15252/emmm.201606198 (PMC5009810; doi:10.15252/emmm.201606198)
Supplement: Supplementary file 4 — Source Data for Figure 3 [file EMMM-8-1052-s003.pdf]

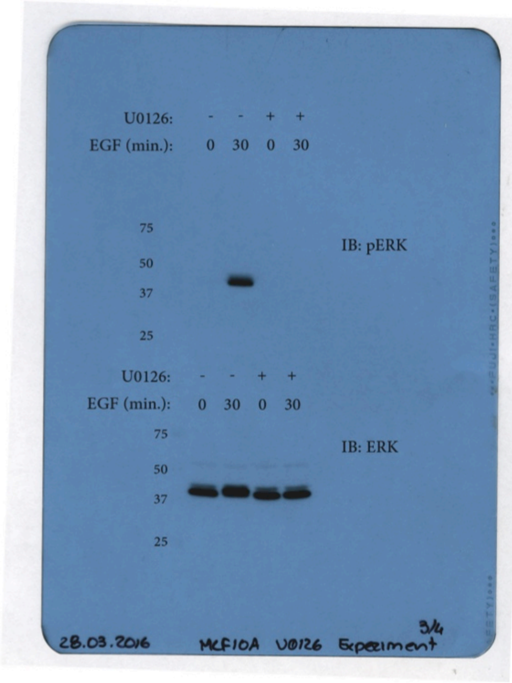

The ERK pathway mediates the effect of EGF on LIMT, which normally inhibits mammary cell migration and invasion. MCF10A cells were treated for 30 minutes with U0126 (a MEK inhibitor), or with no agent, and thereafter EGF was added (10 ng/ml) and incubated with cells for additional 30 minutes. Protein extracts were used to assess levels of phosphorylated ERK (upper) and total level of ERK (bottom). The blot was used as the raw data for Figure 3C.
